# Crystal Structure of the Pre-fusion Nipah Virus Fusion Glycoprotein Reveals a Novel Hexamer-of-Trimers Assembly

**DOI:** 10.1371/journal.ppat.1005322

**Published:** 2015-12-08

**Authors:** Kai Xu, Yee-Peng Chan, Birgit Bradel-Tretheway, Zeynep Akyol-Ataman, Yongqun Zhu, Somnath Dutta, Lianying Yan, YanRu Feng, Lin-Fa Wang, Georgios Skiniotis, Benhur Lee, Z. Hong Zhou, Christopher C. Broder, Hector C. Aguilar, Dimitar B. Nikolov

**Affiliations:** 1 Structural Biology Program, Memorial Sloan-Kettering Cancer Center, New York, New York, United States of America; 2 Department of Microbiology and Immunology, Uniformed Services University, Bethesda, Maryland, United States of America; 3 Paul G. Allen School for Global Animal Health, Washington State University, Pullman, Washington, United States of America; 4 Department of Microbiology, Immunology and Molecular Genetics, University of California, Los Angeles, David Geffen School of Medicine, Los Angeles, California, United States of America; 5 Life Sciences Institute and Innovation Center for Cell Biology, Zhejiang University, Hangzhou, Zhejiang, China; 6 Life Sciences Institute, University of Michigan, Ann Arbor, Michigan, United States of America; 7 CSIRO Animal, Food and Health Sciences, Australian Animal Health Laboratory, Geelong, Victoria, Australia; 8 Program in Emerging Infectious Diseases, Duke-NUS Graduate Medical School, Singapore, Singapore; 9 Department of Biological Chemistry, University of Michigan Medical School, Ann Arbor, Michigan, United States of America; 10 Department of Microbiology, Icahn School of Medicine at Mount Sinai, New York, New York, United States of America; 11 California NanoSystems Institute, University of California, Los Angeles, David Geffen School of Medicine, Los Angeles, California, United States of America; Integrated Research Facility at Fort Detrick, UNITED STATES

## Abstract

Nipah virus (NiV) is a paramyxovirus that infects host cells through the coordinated efforts of two envelope glycoproteins. The G glycoprotein attaches to cell receptors, triggering the fusion (F) glycoprotein to execute membrane fusion. Here we report the first crystal structure of the pre-fusion form of the NiV-F glycoprotein ectodomain. Interestingly this structure also revealed a hexamer-of-trimers encircling a central axis. Electron tomography of Nipah virus-like particles supported the hexameric pre-fusion model, and biochemical analyses supported the hexamer-of-trimers F assembly in solution. Importantly, structure-assisted site-directed mutagenesis of the interfaces between F trimers highlighted the functional relevance of the hexameric assembly. Shown here, in both cell-cell fusion and virus-cell fusion systems, our results suggested that this hexamer-of-trimers assembly was important during fusion pore formation. We propose that this assembly would stabilize the pre-fusion F conformation prior to cell attachment and facilitate the coordinated transition to a post-fusion conformation of all six F trimers upon triggering of a single trimer. Together, our data reveal a novel and functional pre-fusion architecture of a paramyxoviral fusion glycoprotein.

## Author Summary

Paramyxoviruses infect host cells through the coordinated functions of two envelope glycoproteins. The G glycoprotein attaches to cell receptors triggering the fusion (F) glycoprotein to execute membrane fusion. The crystal structure of the NiV-F protein has not been reported. Additionally, many molecular details of the virus-cell fusion process remain elusive, including how the higher-energy pre-fusion conformation state of the F glycoprotein is stabilized, or how many copies of the F glycoprotein are required for fusion. This manuscript reports the pre-fusion crystal structure of NiV-F glycoprotein, and a functional hexamer-of-trimers assembly, with six F trimers encircling a central axis. Multidisciplinary data suggested that this assembly plays a role in the stability of the pre-fusion F conformation prior to cell attachment and F-triggering to a post-fusion conformation. Thus this assembly may coordinate this transition in all six F trimers upon triggering of a single trimer during membrane fusion pore formation.

## Introduction

Henipavirus, a relatively recently recognized viral genus in the family Paramyxoviridae, comprises likely over 20 species, including three established species: Hendra (HeV), Nipah (NiV) and Cedar (CedPV) viruses, with HeV and NiV well-recognized as highly pathogenic agents for both humans and animals [1–6]. Henipaviruses have two surface spike glycoproteins. The G glycoprotein attaches to cell surface receptors, and upon receptor binding triggers the F glycoprotein to execute virus-host cell membrane fusion, facilitating viral entry [7–9]. The host cell receptor proteins employed by the henipaviruses are B-class ephrin molecules [10–12]. The henipavirus F glycoprotein is a trimeric class I transmembrane glycoprotein synthesized as a precursor F_0_ that undergoes post-translational cleavage by host cell cathepsin-L within the endosomal compartment, yielding the fusogenic F_1_ and F_2_ subunits held together by a disulfide bond [13–16]. Crystal structures of other paramyxovirus F glycoproteins in both pre-fusion and post-fusion forms have been reported, supporting a model of the F glycoprotein undergoing a transition from a metastable pre-fusion state to a more thermodynamically stable post-fusion state upon activation [17–23]. This transition brings together the viral envelope and host cell membrane to facilitate membrane fusion and viral entry. Additionally, the same viral glycoproteins facilitate viral spread from infected to naïve cells by a similar cell-cell fusion mechanism (syncytia formation). However, many of the molecular details of the membrane fusion process remain elusive.

Paramyxovirus envelope-host cell membrane fusion likely shares common features with other types of viral and cellular membrane fusion processes, such as influenza virus entry, and synaptic vesicle fusion within neuronal cells. While influenza entry is mediated by viral glycoprotein trimers, synaptic vesicle fusion is triggered by SNARE (soluble N-ethylmaleimide–sensitive factor attachment glycoprotein receptor) molecules that functionally resemble the F glycoprotein oligomers. It has been suggested that at least three hemagglutinin trimers are required for influenza virus entry [24, 25]. It has also been demonstrated that at least three copies of SNAREpins are required for keeping the nascent fusion pore open long enough to ensure efficient neurotransmitter release [26, 27]. However, cooperation of multiple fusion proteins (F glycoproteins) in the paramyxovirus entry process has not yet been demonstrated. Here we report a 3.4 Å crystal structure of the NiV-F glycoprotein in its pre-fusion form. Interestingly, the structure reveals a hexameric pre-fusion assembly consisting of six copies of F glycoprotein trimers encircling a central axis. Electron tomography and structure-based studies in the context of cell-cell fusion and virus-entry further support the functional relevance of the hexamer-of-trimers assembly. Our findings suggest a cooperative F glycoprotein trimer activation model in the NiV entry process, providing insight into the viral-cell and cell-cell membrane fusion mechanisms.

## Results

### Structure of the NiV-F protein trimer

The extracellular region of the NiV-F glycoprotein (residues 1–488) was expressed and purified as described previously [28, 29]. A GCNt helical bundle motif was fused to the C-terminus of the F glycoprotein to stabilize it in the pre-fusion conformation. After successful crystallization and diffraction data collection, the crystal structure of this construct was determined at 3.4 Å resolution by molecular replacement, using the PIV5 pre-fusion F glycoprotein structure (PDB ID 2B9B) as a search model [20], and was refined to R_work_/R_free_ of 23.0/24.8% (for details see Materials and Methods).

**Fig 1:**
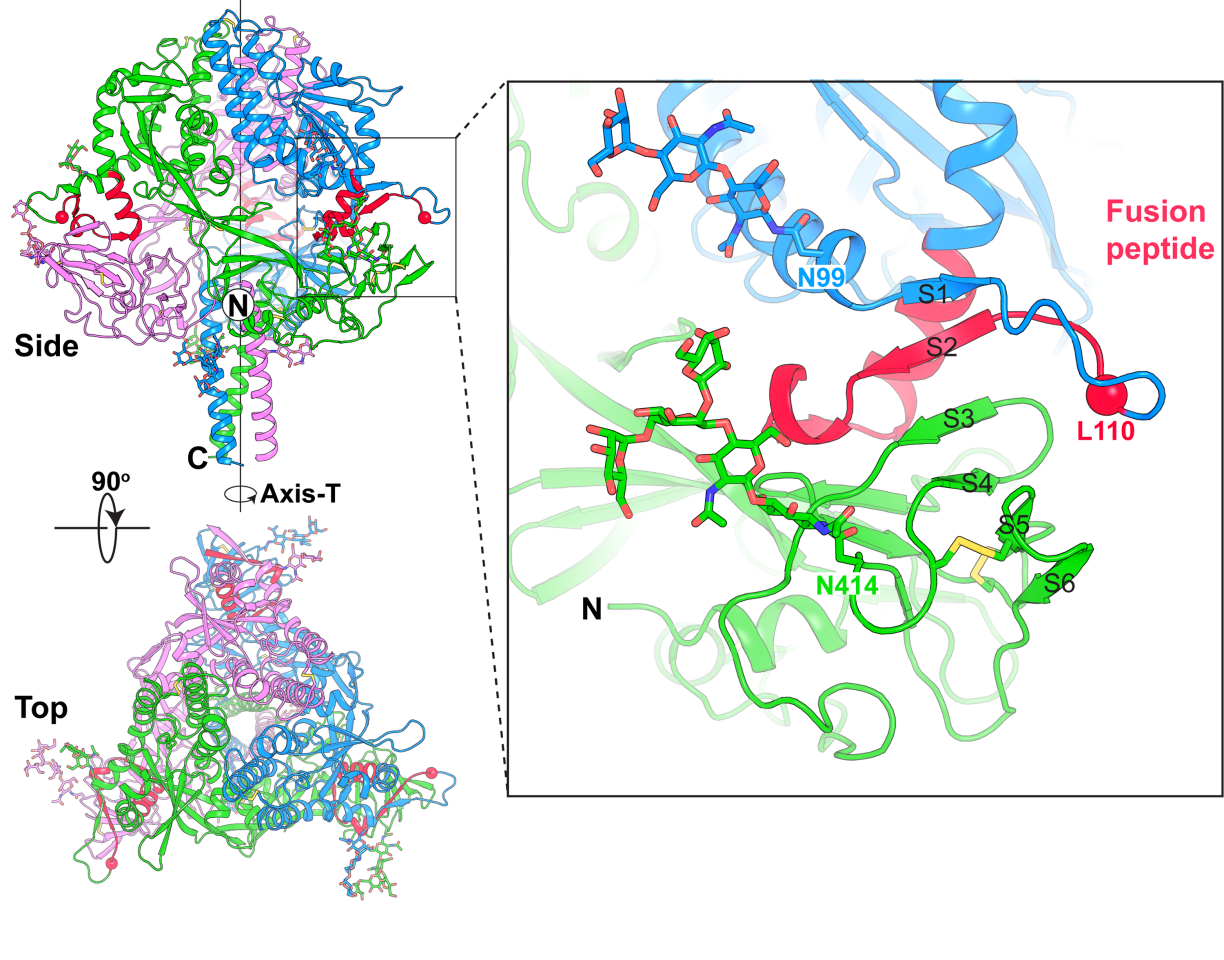
Structure the NiV-F trimer. The NiV-F trimer has a tree-like overall shape with the three copies of the F glycoprotein (colored in blue, green and violet) twined around a central axis (Axis-T). The fusion Peptide (FP) is colored in red. The NiV-F trimer is shown viewed from side, and from the top. Glycosylation moieties and disulfide bonds are shown as sticks. The inset shows a close-up view of the FP, which is located at the N-terminus of the F_1_ subunit, and docks into a groove formed by the F_2_ subunit of a neighboring F molecule within the trimer. Furthermore, the C-terminus of F_2_ and N-terminus of FP fold into a β-hairpin conformation, forming a continuous β-sheet (strands S1-S6) with the F_1_ subunit, which fixes the position of FP and stabilizes the pre-fusion state. The N-terminal FP residue, L110, is shown as a red sphere.

The NiV-F trimer has a “tree-like” overall shape, with the three copies of the F glycoprotein twined around a central axis (Axis-T) that is parallel to the C-terminal helical bundle (Fig 1). The fusion peptide (FP), residing in the N-terminal segment (residues 110–122) of the F_1_ subunit, is docked into a groove formed by the F_1_ subunit of a neighboring F molecule within the trimer. Furthermore, the C-terminus of F_2_ and N-terminus of the FP fold into a β-hairpin (S1-S2), that forms a continuous β-sheet with β-strands S3-S6 in the F_1_ subunit, thus securing the position of the FP and stabilizing the pre-fusion state (Fig 1, inset). The cathepsin-L cleavage site (R109-L110), located on the tip of the β-hairpin S1-S2, is easily accessible for cleavage in the F trimer due to its surface exposure and structural flexibility. The C-terminus of the NiV-F construct (after D482) and the GCN tag are not visible in our structure, suggesting that these regions are disordered.

The structure of the NiV-F glycoprotein most closely resembles the pre-fusion structure of the PIV5 F [20] glycoprotein and the two can be superimposed with Rmsd of 2.5 Å between 1160 Cα atoms (Fig A in S1 Text). This similarity is in line with the 29% identity in their primary sequences. This similarity also suggests that these F glycoproteins undergo a similar pre- to post-fusion transition. Interestingly, the β-hairpin (S1-S2) adopts a conformation similar to that of the cleaved activated (CA)-PIV5 pre-fusion F glycoprotein [30], rather than of the uncleaved form [20] (Fig A in S1 Text, inset). The NiV-F glycoprotein was expressed in human (HEK293) cells, and N-linked carbohydrate moieties were modeled in the known four utilized N-glycosylated sites [31–33] N99, N414, N465 & N485 (Fig 1).

### Hexamer-of-trimers NiV-F assembly

**Fig 2:**
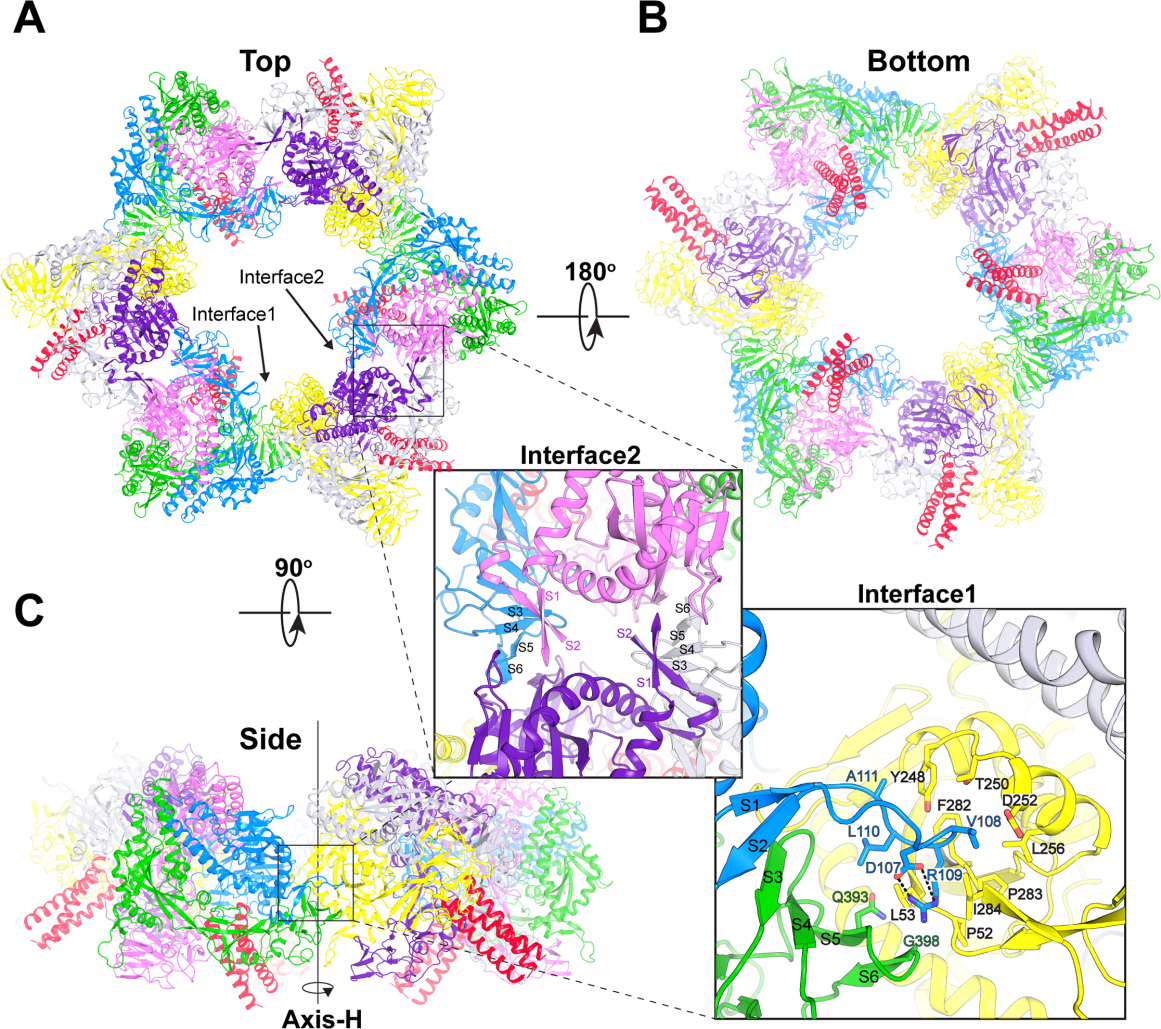
Hexameric Assembly of NiV-F glycoprotein trimers. The six NiV-F copies are colored in blue, green, pink, yellow, grey and purple, respectively. The C-terminal helical bundles of the NiV-F trimers are colored in red. Six NiV-F trimers assemble into a hexagonal ring around a three-fold crystallographic symmetry axis. The hexameric assembly of NiV-F is presented in cartoon views from the top (**A**), bottom (**B**) and side (**C**). The inset in panel C shows a hydrophobic patch of the hexameric interface between two neighboring NiV-F trimers (interface 1). Specifically, the cathepsin-L cleavage site-containing loop of one F trimer inserts into a hydrophobic pocket of the adjacent F trimer. Residue R109 is embedded into a pocket defined by P52, L53, Y248, T250, L256, F282, P283 and I284. The surrounding residues V108, A111, Q393 and G398 also contribute to the interaction. In the two interface inserts, the blue, green, pink, and purple text marks residues in the monomers of the corresponding colors.

NiV-F crystallized in the H3 space group, with two F trimers in each crystallographic asymmetric unit (AU), which, together with the other four counterparts in the two neighboring AUs, form a hexagonal arrangement of six F trimers surrounding a central axis (Fig 2). The Axis-Ts of the six F glycoprotein trimers form alternative 45° and 135° angles to the hexagonal axis (Axis-H). The C-terminal helix-bundles of the six trimeric F glycoproteins are all in the same face of the hexameric assembly, with three of them pointing inwards, and the other three pointing outwards. Such an arrangement renders the maximum contact area between neighboring F trimers due to their “tree-like” shape and surface glycan arrangements.

The observed hexameric assembly is generated via two distinct F-trimer/F-trimer interfaces as indicated on Fig 2, burying approximately 1200 Å^2^ and 1100 Å^2^ surface area, respectively, on each interacting F trimer. Interface 1 (Fig 2C, inset) is mainly between the S1-S6 β-sheet and a hydrophobic patch on the surface of the adjacent F trimer. Intriguingly, the core of interface 1 is formed by insertion of the tip of β-hairpin S1-S2 into a hydrophobic cavity. Specifically, the side chain of R109 is embedded into a groove defined by residues P52, L53, Y248, L256, F282, P283 and I284 of the adjacent F trimer. R109 is further stabilized by a hydrogen bond between its main chain nitrogen and the hydroxyl group of Y248. The positive charge of R109 is neutralized by the neighboring D107. Residues L108, L110 and A111, located on the tip of β-hairpin S1-S2, together with Q393 and G398, located in β-strand S5 and S6, contribute additional hydrophobic interactions. Interface 2 (Fig 2A, inset) is composed of two similar contact regions involving multiple hydrogen bonds and van der Waals’ interactions. In each of these regions, β-sheet S1-S6 on one F trimer interacts with the surface of the adjacent F trimer. The tip of β-hairpin S1-S2 is not visible in the electron density at interface 2, presumably because of its flexibility. The two interfaces in the hexameric assembly would stabilize the pre-fusion F conformation by burying β-sheet S1-S6, and preventing separation of the F_1_ and F_2_ subunits.

### Oligomeric hexamer-of-trimer state of NiV-F in solution and on virus-like particles (VLPs)

**Fig 3:**
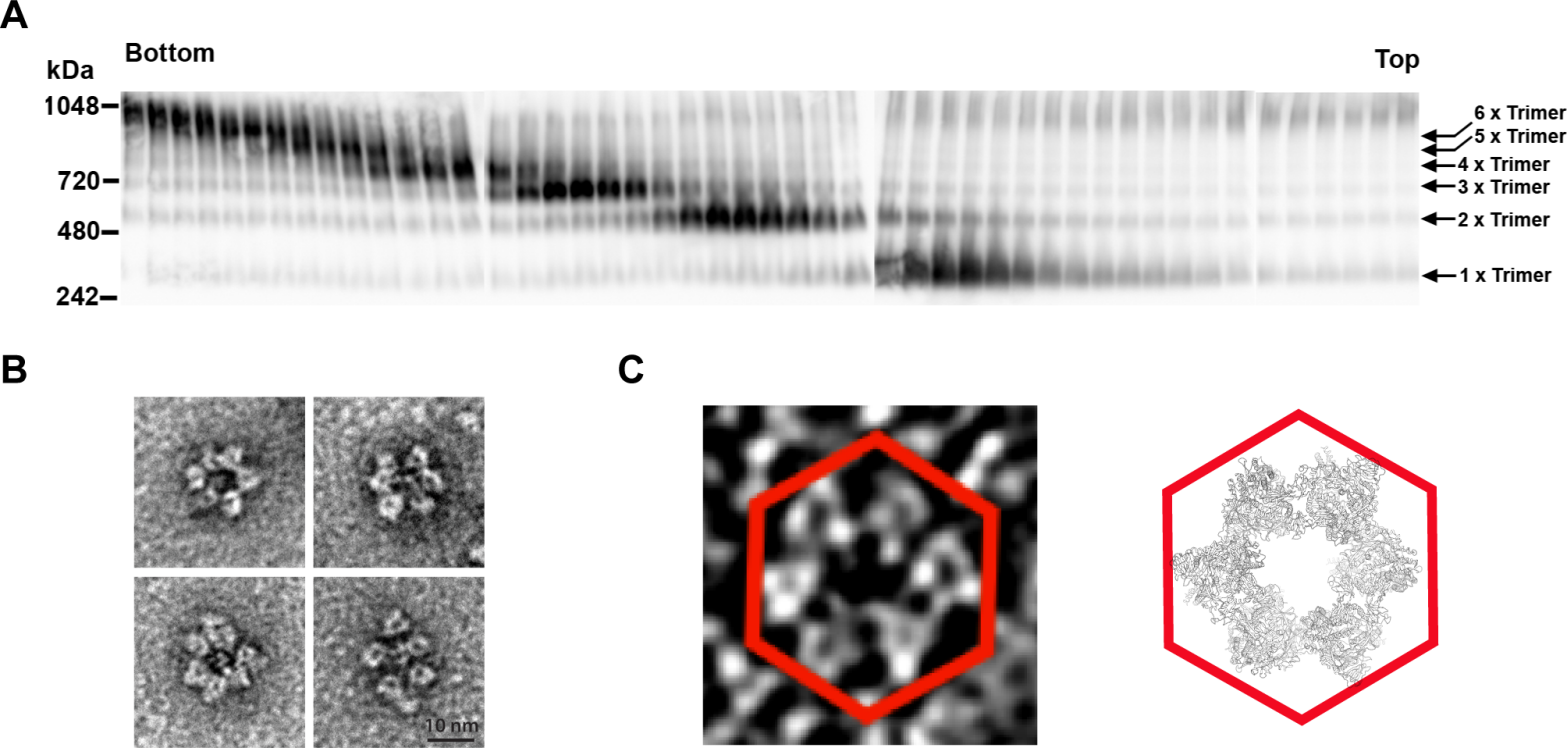
Formation of F hexamers-of-trimers in solution. (**A**) 100μg of 50 mg/mL sF cross-linked with 0.08% glutaraldehyde was subjected to 10–25% sucrose gradient ultracentrifugation and fractionated. 10μL of each fraction was analyzed on Blue Native PAGE followed by western blotting. F oligomers were probed by monoclonal mouse anti-F specific antibody. The bottom and top gradient fractions are indicated. Arrows indicate higher-order oligomers formed from F trimers. (**B**) Hexameric sF assemblies imaged with negative stain EM of pooled fractions 1–6 from panel (A). It shows the cross-linked NiV-F particles boxed out from raw images. The majority of NiV-F oligomers appear as hexamers-of-trimers. (**C**) A small density slab (7.4 nm thick) from an electron tomogram of NiV-F decorated VLP showing the hexameric arrangement of the NiV-F spikes on the VLP. The individual NiV-F spikes are ~8–10 nm long with a ~1–2 nm stalk, consistent with the electron microscopy studies of soluble PIV5-F in its pre-fusion state [59]. The hexameric assembly revealed in NiV-sFGCNt crystal structure was shown next to it as reference.

We next investigated the oligomeric state of NiV-F in solution. Gel-filtration assays indicated that at low concentrations (~1–2 mg/ml) the NiV-sF protein exists as a simple trimer in solution [28]. However, previous cryo-EM studies documented that the paramyxovirus F glycoprotein was densely packed in distinct patches on the virus surface [34], which represent an enriched, high protein concentration 2-D environment. Therefore, purified soluble NiV-F was concentrated to simulate physiological conditions and evaluated oligomer formation using a crosslinking assay. The results are presented in Fig 3A, indicating that higher-order oligomers of NiV-sF, including the apparent molecular weight equivalent of hexamers-of-trimers (top arrow), are indeed formed in solution at high concentrations (~20–45 mg/ml). No oligomers larger than hexamers-of-trimers were observed. Additionally, although the majority of oligomers were not hexamers-of-trimers, but lower-order oligomers, it is likely that cross-linking is not sensitive enough to capture every single hexamer-of-trimers.

Furthermore, examination of the cross-linked species by negative stain EM revealed distinct oligomeric forms including hexamers-of-trimers (Fig 3B), which were the predominant oligomeric form (see Fig B in S1 Text). These oligomers appear somewhat non-uniform and varying in appearance, suggesting that the presence of the lipid membrane and additional steric constraints along the envelope surface may be required for stabilization of the hexamer-of-trimer architecture. To examine this hypothesis we employed electron tomography (ET) to visualize the arrangement of NiV-F in a native membrane environment (Fig 3C). In agreement with both the crystal structure and EM visualization of cross-linked NiV-F, the majority of F trimers were observed as hexamers-of-trimers (in addition to single F trimers–see Fig B in S1 Text) on viral-like particles (VLPs) with full-length F. Moreover, interactions between hexamers-of-trimers were observed, with one trimer being part of more than one hexamer-of-trimers, sometimes resembling a soccer ball arrangement. Although one cannot exclude artifacts due to dehydration during ET and/or heavy metal deposition during negative staining of the VLPs, the shapes and sizes of the observed NiV-F spikes and spike assemblies are fully consistent with the structures of the pre-fusion F trimers, the crosslinking results, and the hexamers-of-trimers observed by X-ray crystallography.

### Hexameric interface mutations affect cell-cell and viral-cell membrane fusion

Next, to evaluate whether there is any functional relevance for the NiV-F hexamer-of-trimer assembly, structure-based targeted mutations were designed to either destabilize (L53D and V108D) or stabilize (R109L and Q393L) the hexameric interfaces. Residues L53, V108 and R109 are among the key components in the formation of the hexameric interface 1 hydrophobic core (Fig 2C, inset); while residue Q393, located in β-sheet S5 (insets of Fig 2A and 2C), is involved in forming both interfaces 1 and 2. L53D, for example, would abrogate the hydrophobic groove in interface 1, while V108D would not only decrease the local hydrophobicity, but also create a repulsive electric force with D252, and weaken interface 1. On the other hand, the R109L substitution would favor embedding the side chain of this residue in the hydrophobic groove of the adjacent F trimer. Similarly, Q393L would not only enhance the hydrophobicity of interface 1, but would also form favorable van der Waals interactions with L53 and I284 of the adjacent F trimer in interface 2.

**Fig 4:**
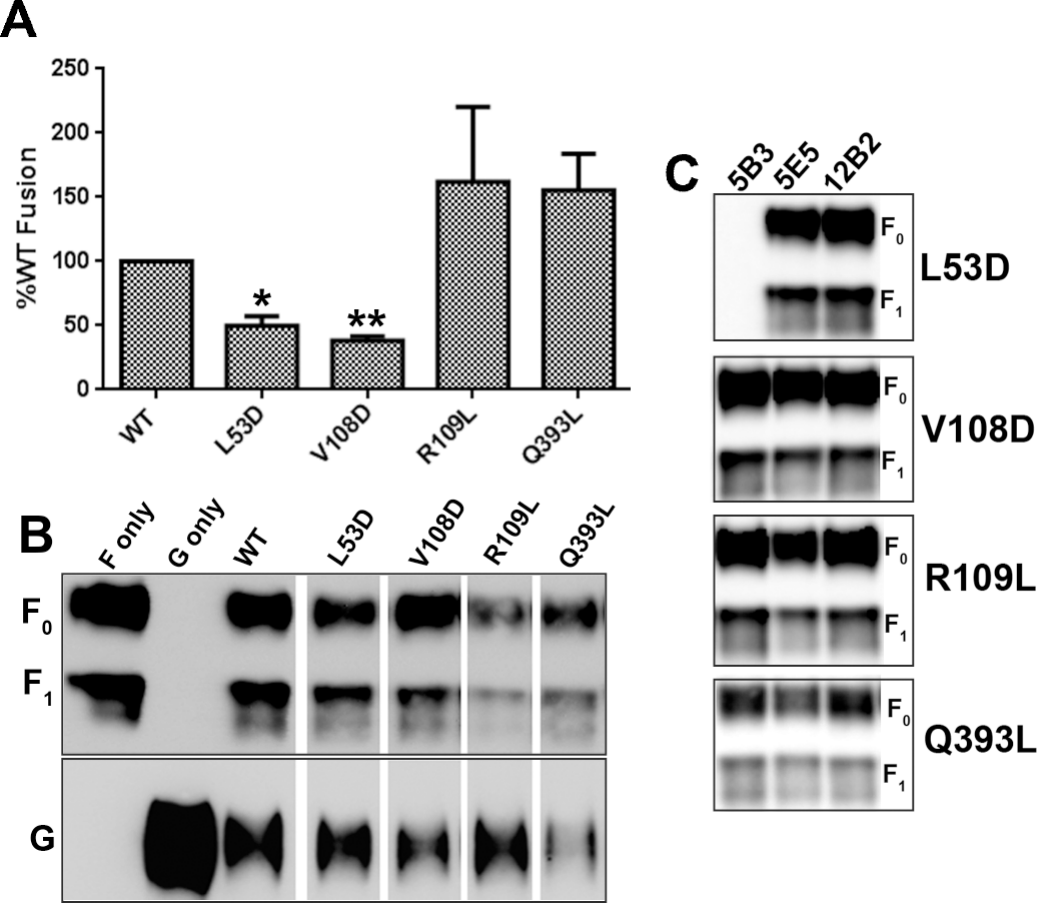
Cell-cell fusion and mAb binding activities of NiV-F mutants. Four mutants of NiV-F within the trimer-trimer interface were tested for their ability to promote cell-cell fusion when co-expressed with NiV-G in a β-Gal reporter cell-cell fusion assay using HEK293T cells as the target population. (**A**) The data shown are the mean percentage of WT fusion levels measured for each mutant calculated from four separate experiments. The data was normalized for cell surface expression of WT F measured by flow cytometry, using a F-specific mAb that recognizes total F surface expression. The bars represent the range from multiple experiments. WT: wild type F. Statistical analysis probes activity deviation relative to WT. *: p<0.01; **: p<0.001; p = 0.3668 for R109L; p = 0.1488 for Q393L. (**B**) Expression of NiV-F mutants and NiV-G in HeLa USU cells from (A). Equal amount of remaining cells from fusion were lysed and clarified by centrifugation followed by immunoprecipitation by polyclonal rabbit anti F and G serum and protein G Sepharose. The precipitated products were analyzed on SDS PAGE followed by western blotting. F and G were probed by monoclonal mouse anti F (upper panel) and G (lower panel) specific antibody. (**C**) Cleared lysates of NiV-F mutants expressed in 293T cells were immunoprecipitated with 3 different competition groups of neutralizing anti F mAb as indicated. All 3 mAbs efficiently immunoprecipitated WT F. The precipitated complexes were analyzed on SDS-PAGE followed by western blotting and F was probed using polyclonal rabbit anti-F antisera.

These four F mutants were evaluated first in a HeLa-USU/HEK293T cell-cell fusion assay (Fig 4A and 4B). Remarkably, the mutations predicted to impair the hexameric interfaces reduced the cell-cell fusion efficiency (P<0.01), while the mutations predicted to stabilize the hexamer-of-trimer interfaces tended to enhance cell-cell fusion efficiency, in comparison to wild-type (wt) NiV-F. The normalized fusion levels shown in Fig 4A already take into account glycoprotein cell surface expression levels. Polyclonal anti-F specific antibody and conformation-specific monoclonal antibodies (mAbs 5B3, 5E5, 12B2) were used to confirm that these mutations in the hexameric interfaces did not affect normal F processing and the overall folding and structure of the F glycoprotein (Fig 4B and 4C). Residue L53 is also a critical residue within the epitope recognized by mAb 5B3 (Chan and Broder, manuscript in preparation), as revealed by its defective binding to the 5B3 mAb (Fig 4C).

**Fig 5:**
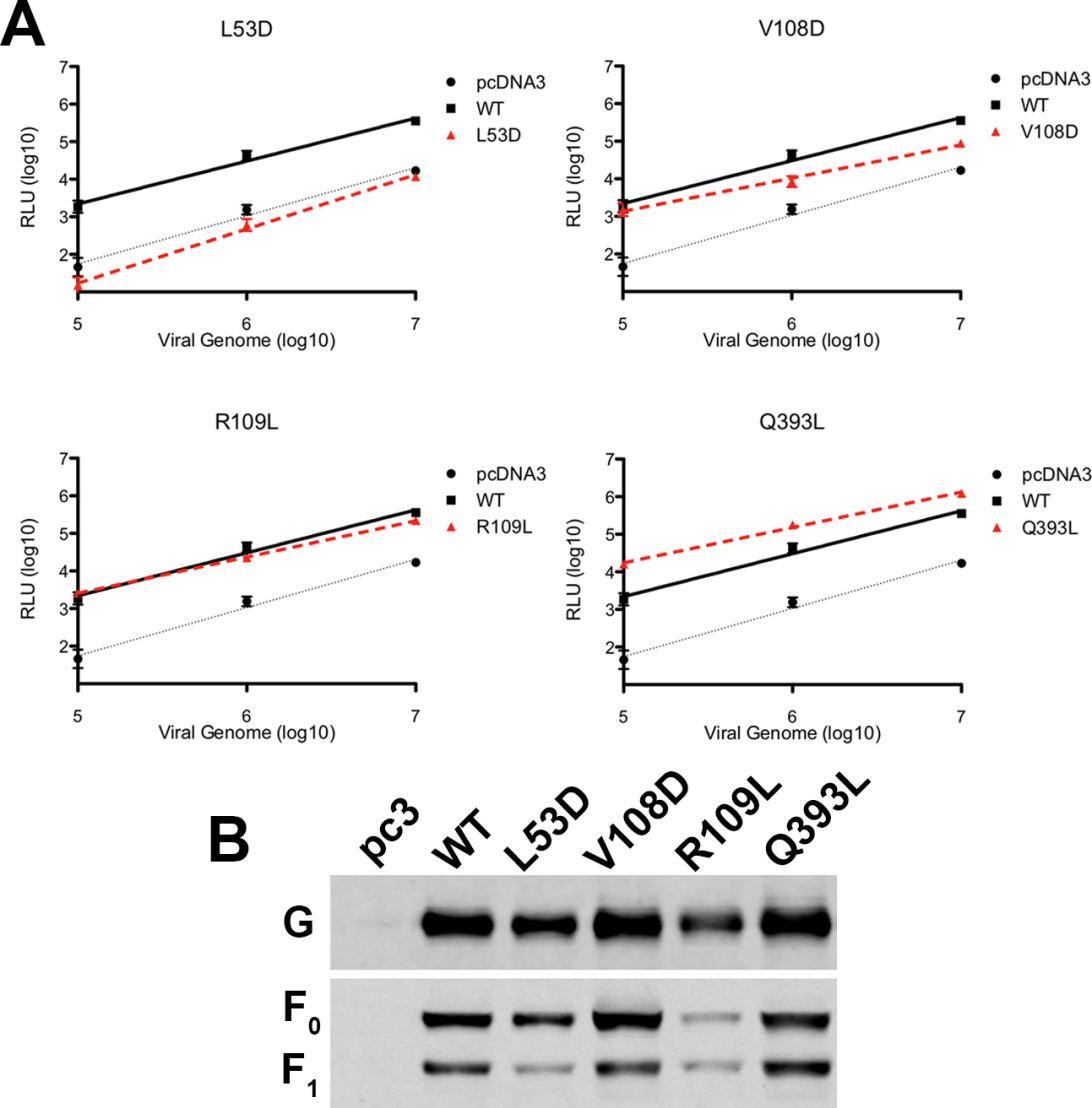
Viral entry is affected by mutations in the hexameric interface. (**A**) Relative entry levels of NiV/VSV-rluc virions containing wt NiV-G and wt NiV-F (solid black line) or mutant L53D, V108D, R109L or Q393L NiV-F (dotted red line). Vector alone (pcDNA3)/VSV is shown as a dotted gray line. RLU for lysates of infected Vero cells were quantified 18–24 h post infection and plotted against the number of viral genomes/ml over 3 logs of viral input. Data shown are averages ± SEM from at least three independent experiments (n = 3). (**B**) Representative Western blot analysis of NiV/VSV-rluc virions shown under Fig 5A. 4x108 NiV/VSV pseudotyped virions (genome copies) were separated by denaturing 10% SDS–PAGE and probed against NiV-G (rabbit anti-HA, Bethyl) and NiV-F (mouse anti-AU1, Covance).

To confirm the importance of the hexamer-of-trimer assembly and interfaces for virus-host cell membrane fusion, the NiV-F mutants were next tested in the context of virion entry. Specifically, a well characterized pseudotyped virus entry assay [31, 35] was used to compare the entry efficiencies of viral particles containing either wt or mutant NiV-F over several logs of viral input. The results (Fig 5) were fully consistent with the results derived from the cell-cell fusion assay. It should be noted that while R109L showed the same viral entry levels as WT F, the overall level of incorporation of R109L into VSV particles was significantly lower than WT F levels, suggesting that this mutant indeed supports higher entry efficiency than the WT F glycoprotein.

## Discussion

**Fig 6:**
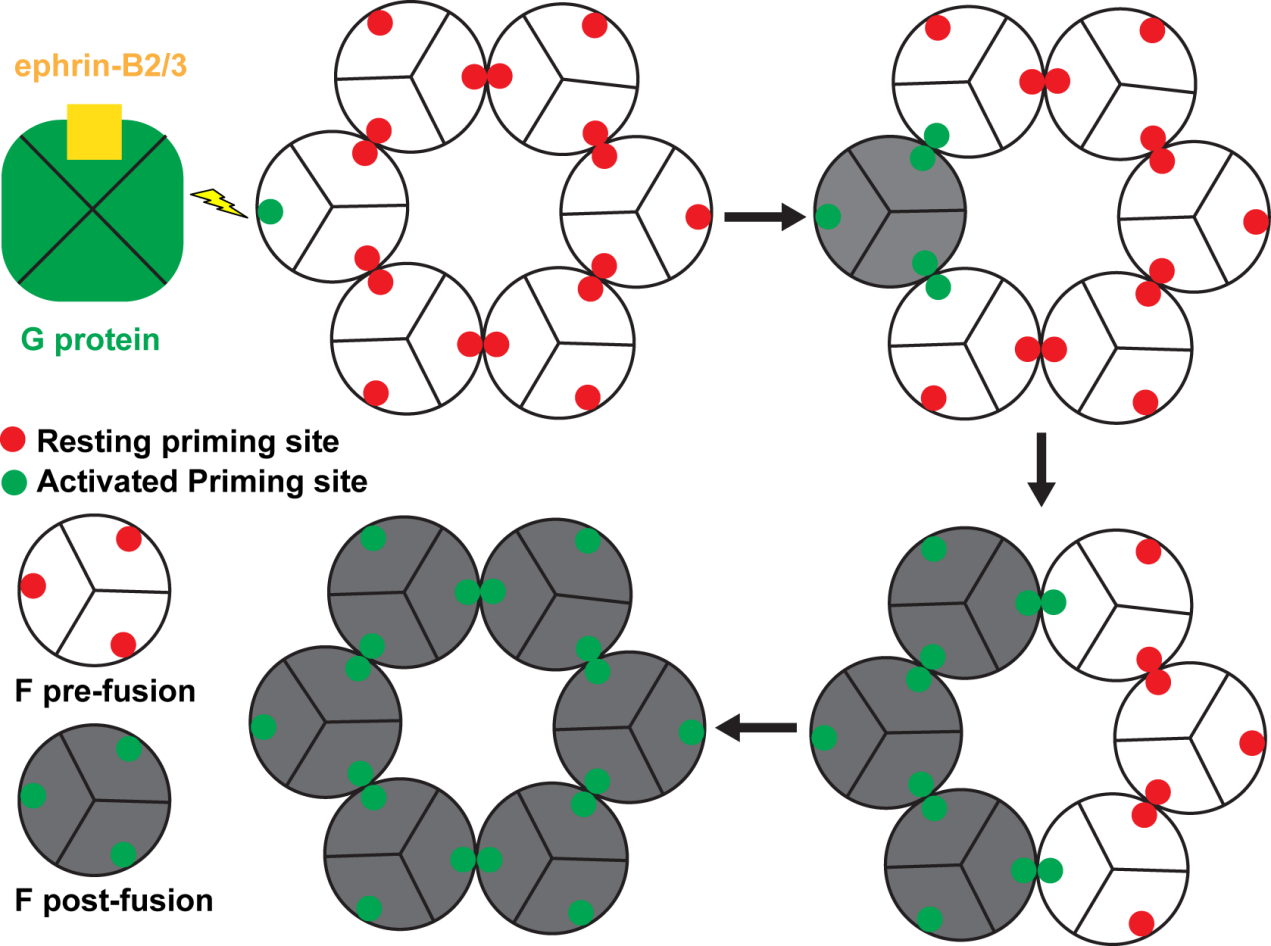
Schematic representation of the proposed NiV-F activation model. Upon viral attachment, the ephrin receptor-mediated re-arrangements of NiV-G exert a triggering disturbance at an exposed “priming site” (presumably via direct G-F priming-site interactions) that is sufficient to activate one F glycoprotein trimer and trigger its transition from a pre-fusion to a post-fusion conformation. The transformation of a single F trimer within the hexameric assembly would disrupt its interactions with both of its neighbors, unlocking their “priming sites” and facilitating their pre-to-post fusion transitions. Thus a single ephrin/NiV-G/F trimer interaction would result in the synergetic switch from a pre-fusion to a post-fusion conformation in all six F trimers within the hexameric assembly. The resulting eighteen copies of the six-helical post-fusion F bundle would form a stable fusion pore allowing virus entry into the host cell.

A synergetic NiV-F activation and a cooperative fusion-pore opening model can be deduced from our results as depicted in Fig 6. Significant conformational changes and movement of the FP region are thought to occur during F protein transition from pre-fusion to post-fusion states. The β-sheet S1-S6 structure (insets of Figs 1, 2A, 2C and S1 Text) partially sequesters the FP, therefore stabilizing the pre-fusion F conformation. Disruption of this β-sheet would free the FP and facilitate the energetically favorable transition to a post-fusion conformation, providing an efficient way to trigger F protein activation. In the hexameric F assembly, β-sheet S1-S6 is buried in the hexameric interface and, therefore, the pre-fusion F conformation is stabilized. On the other hand, disturbance or dissociation of the hexameric assembly would apply mechanical force on the interface-forming β-sheet S1-S6 triggering F protein activation. The residues on the tip of β-hairpin S1-S2, as well as the hexameric interface-forming residues on β-strands S3-S6, are therefore crucial for the regulation of NiV-F activation and fusion efficiency in this model, and we refer to these sites as “priming sites”. Each F-trimer contains 3 priming sites–two of which are buried within the hexamer interfaces, while the third is available for interaction with (and priming by) the NiV-G/ephrin complex (Fig 6).

Consistent with this model, several of the residues in this “priming site” have been previously shown to mediate the viral fusion-attachment (F-G) protein interactions in the PIV5 virus [36]. Upon viral attachment, the ephrin-mediated re-arrangements of NiV-G would exert a triggering disturbance at an exposed “priming site” (presumably via direct G-F priming-site interactions). This triggers the F glycoprotein trimer to undergo a conformational change from a pre-fusion to a post-fusion configuration. Upon activation by a NiV-G/ephrin complex, the contacted F trimer would not only destabilize the pre-fusion state in its interacting neighbors, but could also directly facilitate an alteration in their “priming sites,” triggering a conformation switch in the rest of the hexamer-of-trimer assembly. Thus, a single ephrin/NiV-G/F trimer interaction would result in the synergetic switch from a pre-fusion to a post-fusion conformation in all six F trimers within the assembly (Fig 6). The resulting eighteen copies of the post-fusion F protein (forming eighteen six-helix bundles) is more likely to pull the two membranes together than triggering of a single F trimer alone. The combination of biochemical and microscopical data (Fig 3), and functional cell-cell fusion (Fig 4), and viral entry (Fig 5) data, is consistent with this model.

The necessity for multiple fusion glycoproteins acting together to affect membrane fusion has been most clearly demonstrated for the SNAREpins (composed of v- and t- SNAREs) during the neurotransmitter cargo-releasing fusion process in synapses [26, 27]. Several lines of evidence suggest similar requirements for various enveloped viruses, including influenza, HIV, baculovirus, Semliki Forest virus, and vesicular stomatitis virus [24, 25, 37–43]. Multiple copies of fusion glycoproteins around the entry site would provide a radial force around the newly fused lipid bilayers that cooperatively stabilizes and opens up the nascent fusion pore, allowing efficient virus entry into the host cell. The core fusion machinery for NiV entry consists of a single glycoprotein, F, which localizes at the viral envelope prior to viral entry. Furthermore, the attachment and the fusion process are exerted by two separate glycoproteins (G and F, respectively). Therefore, the oligomeric assemblies of F glycoproteins and their synergetic activation triggered by the ephrin/G complexes provide an efficient way to ensure the simultaneous availability of multiple NiV-F helix bundles at the contact zone where G glycoproteins are attracted to the host cell-surface ephrins.

Noteworthy, the cathepsin cleavage site (R109/L110) is accessible in the trimer (Fig 1), but fairly buried in interface 1 of the hexameric assembly (Fig 2). So how/when does the cathepsin cleavage occur? F glycoproteins can exist as trimers on the cell surface and on virions in both cleaved and uncleaved forms [44, 45]. Following cathepsin-L cleavage F could shift primarily to the hexamer-of-trimer assembly. Alternatively however, cleavage of F may occur even in the hexamer-of-trimer structural assembly, as the cleavage site is not completely blocked in all hexameric interfaces. In addition, it is likely that R109 and L110 are not the only determinants of F cleavage, as at least F mutant R109 is still cleaved (Figs 4C and 5B). It is clear that F trimers traffic to the plasma membrane before cleavage and then are cycled back into the cell where cleavage occurs, and then recycled back to the cell surface, and both cleaved and uncleaved F exist inside and on the surface of expressing cells [14, 35, 46]. It is also possible that both cleaved and uncleaved F monomers co-exist within a trimer and/or within the hexamer-of-trimer assembly. Furthermore, it is also possible that a hexameric structure is not static and that trimers may form and disassemble from higher order oligomeric forms allowing for cleavage to occur.

Further experimentation is required to unequivocally establish this mechanistic model. Although evidence in this study suggest that a hexamer-of-trimers assembly is a functional form for the F protein, it remains to be determined whether this is the primary assembly form of F in the presence of G. Furthermore, studies of other paramyxoviruses indicate that the transmembrane regions of the F proteins are critical to stabilize the pre-fusion structure, often requiring a stabilizing GCN4 domain [20, 30]. Consistently, our pre-fusion structure and hexamer-of-trimer assembly observed for NiV-F was a result of addition of a GCN4t domain to replace the F transmembrane and cytoplasmic tail regions. It remains to be determined whether the same structural features will be observed for the full-length F proteins embedded in a cellular membrane. Technical challenges prevent such studies from being performed under current available technologies. However, the prevalence of F hexamers-of-trimers in VLPs containing full-length F without a GCN4t domain, in combination with our functional studies, support our current F pre-fusion and hexamer-of-trimers structural results. In addition, it remains to be determined whether this relatively simple mechanistic model applies to other enveloped viruses. It should be noted that various oligomeric viral fusion-protein assemblies, e.g. containing 3–10 copies, could serve the same purpose in other viral systems by potentially stabilizing the pre-fusion conformation and by facilitating a cooperative transition to the post-fusion conformation in all glycoproteins within the assembly upon transition of a single fusion protein.

## Materials and Methods

### Construct design and glycoprotein expression

The construction, expression and purification of the GCNt stabilized pre-fusion soluble version of the NiV-F glycoprotein (NiV-sFGCNt) had been detailed previously [28, 29]. Briefly, The predicted transmembrane (TM) anchor domain (residues 488–510) and the C-terminal cytoplasmic tail (CT) domain (residues 511–546) of the NiV- F were replaced by the GCNt motif (MKQIEDKIEEILSKIYHIENEIARIKKLIGE) [47] in heptad phase followed by a Factor Xa protease cleavage site (IEGR) and the S-peptide tag. A 293T stable cell line expressing the NiV-sFGCNt was generated. Preparation of the sF glycoprotein from 293T stable cell lines was carried out using serum-free culture conditions and employing a combination of S-protein agarose (EMD Biosciences) affinity column and size exclusion chromatography using a HiLoad 16/60 Superdex 200 preparative grade gel filtration column (GE Healthcare) steps to isolate pure NiV-sFGCNt trimer.

### Crystallization and structure determination

Crystals of NiV-sFGCNt formed after two weeks in 2μl hanging drops by vapor diffusion against reservoir solution (in a 1:1 protein-to-reservoir-solution ratio) containing 0.1 M HEPES pH 7.3, 1% Jeffamine ED-2001 and 1.2 M Sodium Malonate. For cryo-protection, crystals were soaked step-wise in reservoir solution starting from 5% up to 25% glycerol and were flash-frozen in liquid nitrogen. Diffraction data were collected at beamline NE-CAT ID-24 of the Advanced Photon Source at Argonne National Laboratory and processed with HKL2000 program package[48]. The structure was determined by molecular replacement with PDB ID 2B9B (PIV5 pre-fusion F protein) as the searching model and MOLREP program in CCP4 Suite [49]. The structure was refined carefully with grouped B-factor refinement (two B-factors per residue), and non-crystallographic symmetry (NCS) restrains in CNS1.2 [50]. NCS restrains were applied to the six chains of NiV-F in one asymmetric unit. The electron density maps for model building were improved by B-factor sharpening with a value of -70 Å^2^. All model building was performed in Coot [51]. The final structure model was checked by the program PROCHECK [52]. Statistics of data collection and refinement are listed in Table A in S1 Text. Noteworthy, all conserved Cys residues aligned between the NiV and PIV5 F structures.

### Crosslinking assay and Sucrose gradient PAGE

Purified NiV-sFGCNt trimer was concentrated to 50 mg/mL using the Corning Spin-X UF 500μL Centrifugal Concentrator (Corning Inc). Glutaraldehyde was added to the concentrated material to various final concentrations ranging from 0.005% to 0.1% yielding protein concentrations between 40–45 mg/mL. 5μg of cross-linked products were analyzed on Blue Native PAGE (Invitrogen) with Coomassie staining. To separate different oligomeric species of cross linked NiV-sFGCNt trimer, 100μg of the 50 mg/mL sF was cross-linked with 0.08% gluteraldehyde yielding a final protein concentration of 42 mg/mL and the cross linked material was then subjected to 10–25% sucrose gradient ultracentrifugation and fractionated. To obtain the gradient, 6 ml of 10% sucrose was underlaid with 6 ml of 25% sucrose in a polyallomer 14- by 95-mm tube. A linear sucrose gradient was generated using the Biocomp Gradient Master (Biocomp, Frederickton, NB, Canada) at an angle of 81.5° for 2 min 19 seconds at a speed of 14k rpm. The cross linked material was then overlaid on top of the gradient. The gradient was centrifuged at 40,000 rpm for 20 h at 4°C using an SW40 rotor (Beckman Coulter, Inc.). Fractions of ~200 μl each were collected from the bottom to the top of the gradient using a Beckman fraction recovery system and automated fraction collector. To analyze the fractions, 10 μl of each collected fraction was resolved on 3–12% BN-PAGE (Invitrogen) followed by western blotting. F oligomers were probed by monoclonal mouse anti-F specific antibody. Fractions with higher oligomers were pooled, desalted, concentrated and analyzed on Blue Native PAGE using Coomassie staining.

### Negative stain electron microscopy

Samples were prepared using conventional negative staining protocols [53]. Briefly, 3 μL of sample was pipetted onto a glow-discharged carbon-coated grid and stained with 1% (w/v) uranyl formate. Imaging was performed at room temperature with a Morgagni 268(D) transmission electron microscope (FEI Company) at 100kV at a magnification of 30,416x.

### Electron Tomography of VLPs

NiV-F VLPs were produced by expressing NiV-F on 293T cells and then collecting, clearing, and purifying the cell supernatants through 20% sucrose, as previously established [54]. It has also been established that NiV-F can autonomously assemble and produce budding of NiV-F VLPs [55]. 3 μl VLP sample was applied on carbon covered copper grids. After one minute the fluid was absorbed with filter paper and 3 μL urinyl acetate (UA) (2.5%) was applied for 40 seconds. Excess UA was absorbed and the grid was left to dry for 2 minutes before being transferred to a Gatan-626 specimen holder. A Tecnai F20 transmission electron microscope (200kV) (FEI Company, OR) equipped with a field mission gun (FEG), computer-controlled compustage, a TIETZ F415MP 16 megapixel CCD camera was used to obtain a total of 141 tomographic images at tilts from -70 to +70 degrees. Tilt series were collected at 29,000x magnification with an applied defocus of -3 μm with FEI’s Batchtomography package. Tilt series alignment and tomographic reconstruction were performed by IMOD [56].

### Cell-cell fusion assays

Fusion between NiV F and G glycoprotein-expressing effector cells and permissive target cells was measured by a β-Galactosidase (β-Gal) assay that was previously described [57]. Briefly, plasmids encoding WT NiV F or each mutant of F and NiV G at a 1:1 ratio or control/mock transfection were transfected into HeLa-USU effector cells. The transfected cells were then infected with vaccinia virus-encoding T7 RNA polymerase the following day. HEK293T cells served as receptor-positive target cells were also infected with the E. coli Lac Z-encoding reporter vaccinia virus. Cells were infected at an MOI of 10 and incubated at 31°C overnight. Cell-cell fusion reactions were conducted by incubating the target and effector cell mixtures at a ratio of 1:1 in 96-well plates at 37°C. Cytosine arabinoside (40μg/ml) was added to the fusion reaction mixture to reduce nonspecific β-Gal production. Nonidet P-40 was added to 0.5% final concentration at 2.5 hrs, and aliquots of the lysates were assayed for β-Gal at room temperature with the substrate chlorophenol red–D-galactopyranoside (Roche). Assays were performed in triplicate, and fusion results were calculated and expressed as rates of β-Gal activity (change in optical density at 570 nm per minute x 1,000) in VersaMAX microplate reader (Molecular Devices, Sunyvale, CA). Equal amount of leftover envelope glycoprotein expressing effector cells from each fusion reaction was used for evaluation of total F and G expression by immunoprecipitation and cell surface F expression by flow cytometry. For immunoprecipitation, cells were lysed and clarified by centrifugation. The lysates were then subjected to rabbit polyclonal anti-F or -G specific antisera and protein G Sepharose precipitation followed by SDS PAGE and western blot analysis. The blots were probed with F or G specific murine mAbs. For flow cytometry, the envelope expressing cells were washed once with PBS and then incubated with pre-fusion specific F mAb followed by incubation with fluorescein isothiocyanate (FITC)-conjugated polyclonal anti mouse Ab (Cell Signaling Technology, Inc., Danvers, MA). All incubations were in PBS with 3% goat serum and on ice for 1 h. Samples were washed three times with cold PBS between incubations and before being fixed in 1.6% paraformaldehyde and then analyzed on a Beckman Coulter Epics XL flow cytometer. The individual cell fusion reactions mediated by each mutant were converted to percentages of WT fusion activity and normalized with cell surface expression of WT F and each F mutants.

### Pseudotyped virus entry assay

VSV-rLuc pseudotype viruses, harboring NiV-F and–G on their surface, were produced as previously described [31]. All NiV-F (C-terminal AU1-tag) and–G (C-terminal HA-tag) constructs, utilized for the production of the pseudotyped viruses, were cloned in the pcDNA3.1 vector. Viral copy numbers were quantified by real-time PCR [31] and 10-fold serial dilutions of each viral prep were tested on Vero cells for viral entry using a renilla luciferase detection system (Pierce) [58]. Relative light units (RLUs) were plotted against the viral genome copy number per milliliter and analyzed by linear regression using GraphPad Prism as previously described [58].

### Illustrations

All molecular representations were produced with PyMOL (Delano Scientific LLC). Figures were prepared using Adobe Illustrator, Adobe Photoshop.

## Supporting Information

S1 Text (Fig A) Comparison of the NiV-F trimer structure with these of the cleavage-activated (CA)-PIV5-F, and non-activated PIV5-F. (Fig B) Representative raw NiV-F EM and VLP-tomography images. (Table A) Crystallographic data collection and model refinement statistics. (DOC)
