# Supplementary material for: Crystal Structure of the Pre-fusion Nipah Virus Fusion Glycoprotein Reveals a Novel Hexamer-of-Trimers Assembly
Source: PLoS Pathog. 2015 Dec 8;11(12):e1005322. doi: 10.1371/journal.ppat.1005322 (PMC4672880; doi:10.1371/journal.ppat.1005322)
Supplement: S1 Text — (DOC) [file ppat.1005322.s001.doc]

Supporting Information (S1 Text):

**Fig. A. Comparison of the NiV-F trimer structure with these of the cleavage-activated (CA)-PIV5-F, and non-activated PIV5-F.** Superimposition of three F-trimer structures (NiV-F, in marine; CA-PIV5-F, in yellow; and PIV5-F, in grey). The structures are overall quite similar, with the exception of the conformations of the cleavage loops, which are highlighted in the inset. The conformation of the cleavage-site region of the non-cleaved PIV5-F is different from the other two structures (is more flexible), while in both NiV-F and CA-PIV5-F this region folds into a β-hairpin, forming a continuous β-sheet with the F_1_ subunit.

**Fig. B. Representative raw NiV-F EM and NiV-F VLP-tomography images. A.** A representative raw EM image of cross-linked soluble NiV-F embedded in negative stain. **B.** Tomography image of the surface of a NiV-F-containing VLP. Three NiV-F hexamers are highlighted with red circles. For both soluble NiV-F and NiV-F VLPs, the vast majority of F trimmers were observed in hexamer-of-trimer arrangements.

**Table A. Crystallographic data collection and model refinement statistics.**

####
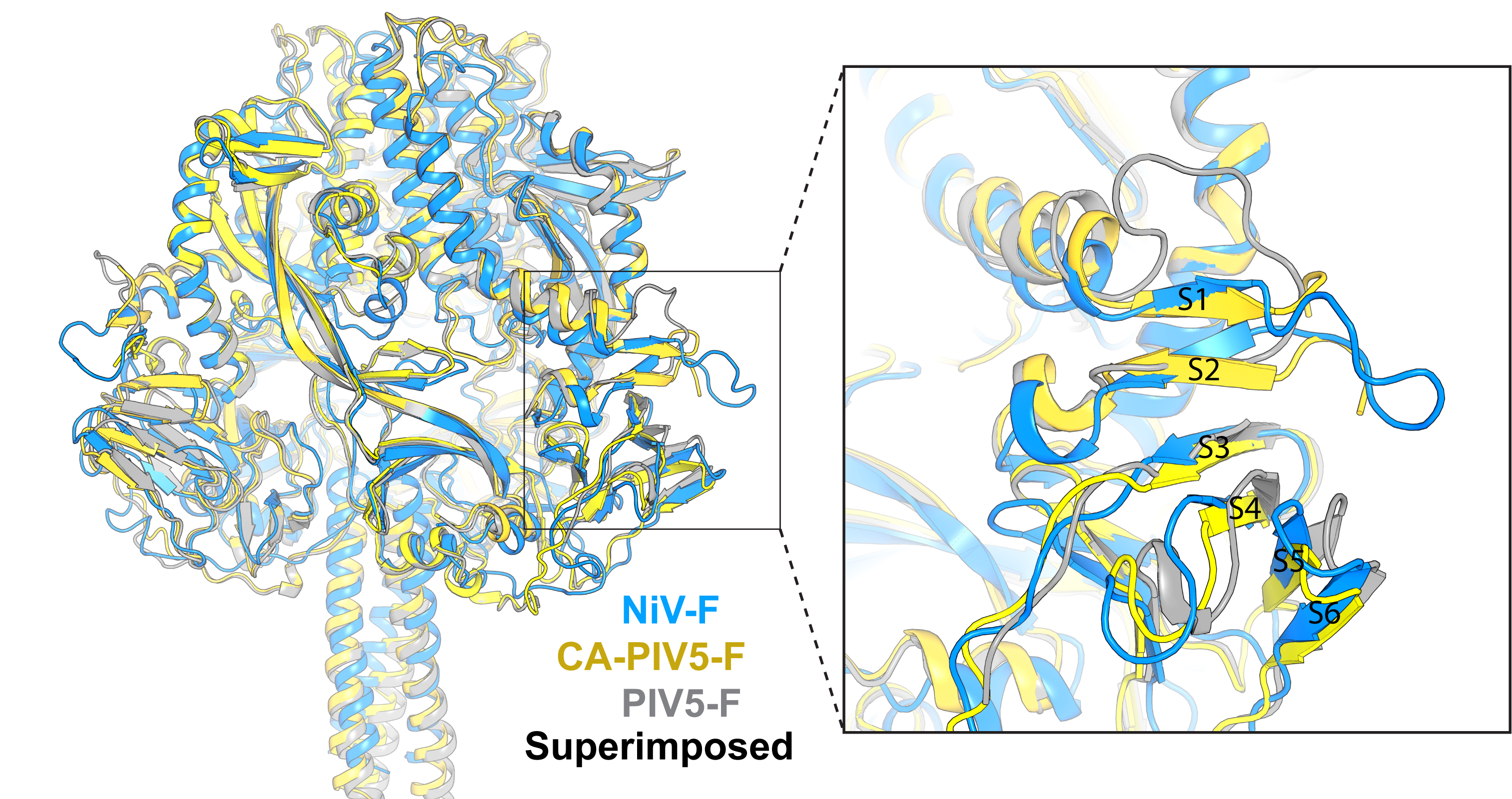


**Fig. A.**


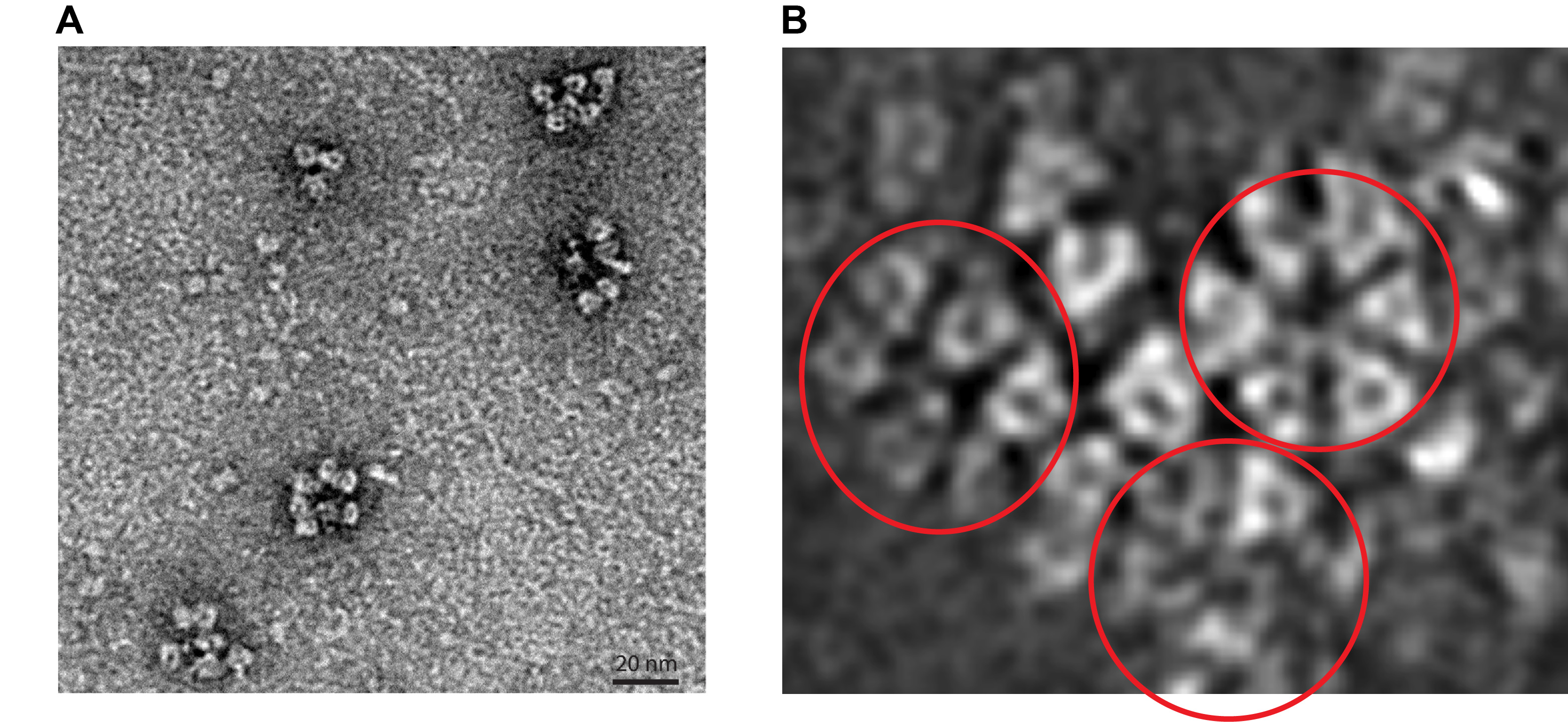


**Fig. B.**

**Table A.**

|  | NiV-F |
| --- | --- |
| Wavelength (Å) | 0.9792 |
| Resolution range (Å) | 50 - 3.35 (3.47 - 3.35) |
| Space group | R3 :H |
| Unit cell | 355.75 355.75 168.859 90 90 120 |
| Total reflections | 664452 |
| Unique reflections | 112120 |
| Multiplicity | 5.9 (5.5) |
| Completeness (%) | 99.36 (93.93) |
| Mean I/sigma(I) | 13.49 (2.41) |
| Wilson B-factor | 103.81 |
| R-merge | 0.161 (0.975) |
| R-work | 0.2213 (0.4043) |
| R-free | 0.2243 (0.4155) |
| Number of atoms | 21612 |
| macromolecules | 20802 |
| ligands | 810 |
| water | 0 |
| Protein residues | 2790 |
| RMS(bonds) | 0.010 |
| RMS(angles) | 1.52 |
| Ramachandran favored (%) | 86 |
| Ramachandran outliers (%) | 1.1 |
| Clashscore | 29.07 |
| Average B-factor | 123.10 |
| macromolecules | 121.30 |
| ligands | 169.20 |

Statistics for the highest-resolution shell are shown in parentheses.
